# Multiscale modeling of the causal functional roles of nsSNPs in a genome-wide association study: application to hypoxia

**DOI:** 10.1186/1471-2164-14-S3-S9

**Published:** 2013-05-28

**Authors:** Li Xie, Clara Ng, Thahmina Ali, Raoul Valencia, Barbara L Ferreira, Vincent Xue, Maliha Tanweer, Dan Zhou, Gabriel G Haddad, Philip E Bourne, Lei Xie

**Affiliations:** 1 Skaggs School of Pharmacy and Pharmaceutical Sciences, University of California San Diego, La Jolla, CA 92093, USA; 2 Department of Computer Science, Hunter College, The City University of New York, New York City, NY 10065, USA; 3 Department of Biological Sciences, Hunter College, The City University of New York, New York City, NY 10065, USA; 4 Department of Psychology, Hunter College, The City University of New York, New York City, NY 10065, USA; 5 Department of Pediatrics, University of California, San Diego, La Jolla, CA 92093, USA; 6 Department of Neuroscience, University of California, San Diego, La Jolla, CA 92093, USA; 7 Rady Children's Hospital, San Diego, CA 92123, USA; 8 Graduate Center, The City University of New York, New York City, NY 10016, USA

## Abstract

**Background:**

It is a great challenge of modern biology to determine the functional roles of non-synonymous Single Nucleotide Polymorphisms (nsSNPs) on complex phenotypes. Statistical and machine learning techniques establish correlations between genotype and phenotype, but may fail to infer the biologically relevant mechanisms. The emerging paradigm of Network-based Association Studies aims to address this problem of statistical analysis. However, a mechanistic understanding of how individual molecular components work together in a system requires knowledge of molecular structures, and their interactions.

**Results:**

To address the challenge of understanding the genetic, molecular, and cellular basis of complex phenotypes, we have, for the first time, developed a structural systems biology approach for genome-wide multiscale modeling of nsSNPs - from the atomic details of molecular interactions to the emergent properties of biological networks. We apply our approach to determine the functional roles of nsSNPs associated with hypoxia tolerance in *Drosophila melanogaster*. The integrated view of the functional roles of nsSNP at both molecular and network levels allows us to identify driver mutations and their interactions (epistasis) in H, Rad51D, Ulp1, Wnt5, HDAC4, Sol, Dys, GalNAc-T2, and CG33714 genes, all of which are involved in the up-regulation of Notch and Gurken/EGFR signaling pathways. Moreover, we find that a large fraction of the driver mutations are neither located in conserved functional sites, nor responsible for structural stability, but rather regulate protein activity through allosteric transitions, protein-protein interactions, or protein-nucleic acid interactions. This finding should impact future Genome-Wide Association Studies.

**Conclusions:**

Our studies demonstrate that the consolidation of statistical, structural, and network views of biomolecules and their interactions can provide new insight into the functional role of nsSNPs in Genome-Wide Association Studies, in a way that neither the knowledge of molecular structures nor biological networks alone could achieve. Thus, multiscale modeling of nsSNPs may prove to be a powerful tool for establishing the functional roles of sequence variants in a wide array of applications.

## Background

Recent advances in next generation sequencing have generated abundant genetic variants and "omics" data. Together, these extremely large, multidimensional datasets present an exciting opportunity to identify genes, and to predict pathways likely to be involved in diseases and traits. However, these complex data sources plus the broad spectrum of phenotypes, challenge the quest to uncover the genetic, molecular, and cellular mechanisms that underlie phenotypes [1-3]. A major challenge in deciphering the genetic basis of multigenic diseases or traits is to distinguish driver mutations that impact the survival or reproduction of a particular phenotype (e.g., cancer) from passengers that do not confer a selective advantage. Standard genome sequence analysis cannot detect all driver mutations due to difficulties in the estimation of the background mutation rate and underlying genetic heterogeneity of adaptive phenotypes [4,5]. Statistical machine learning techniques (e.g., SNAP [6]) provide an alternate approach by learning from the annotated mutation data. However, the "black-box" nature of machine learning makes it difficult to interpret the novel functional roles of mutations. Parallel to the development of new genotyping and phenotyping techniques, a number of novel computational tools have been developed to integrate and analyze genetic and omics data with the aim of establishing statistical causal relationships between genetic markers, genome-wide molecular signatures, and organismal phenotypes [7-13]. For example, co-expression and Bayesian network models derived from DNA variances and genome-wide transcriptional profiles have been applied to identify causal disease genes [14], cancer drivers [10,15], and master regulators of cancer [16-18]. Although great efforts have been made to address *n*<<*p *problem, where the number of observations *n *(e.g., gene expressions in different conditions) is much smaller than the number of variables or parameters *p *(e.g., all measured genes), the power of these statistics-based techniques is still limited if sample sizes are small. Moreover, the complex phenotype is often associated with interactions among multiple causal genes (epistasis), any of which alone is not sufficient to drive phenotypic change. It is challenging for statistical methods to identify epistasis given the large number of possible interactions. Fundamentally, the "causal" relationships inferred from these methods are mathematical correlations. They may not provide biological insight into the underlying molecular and cellular mechanisms that associate genotypes with phenotypes.

**Figure 1:**
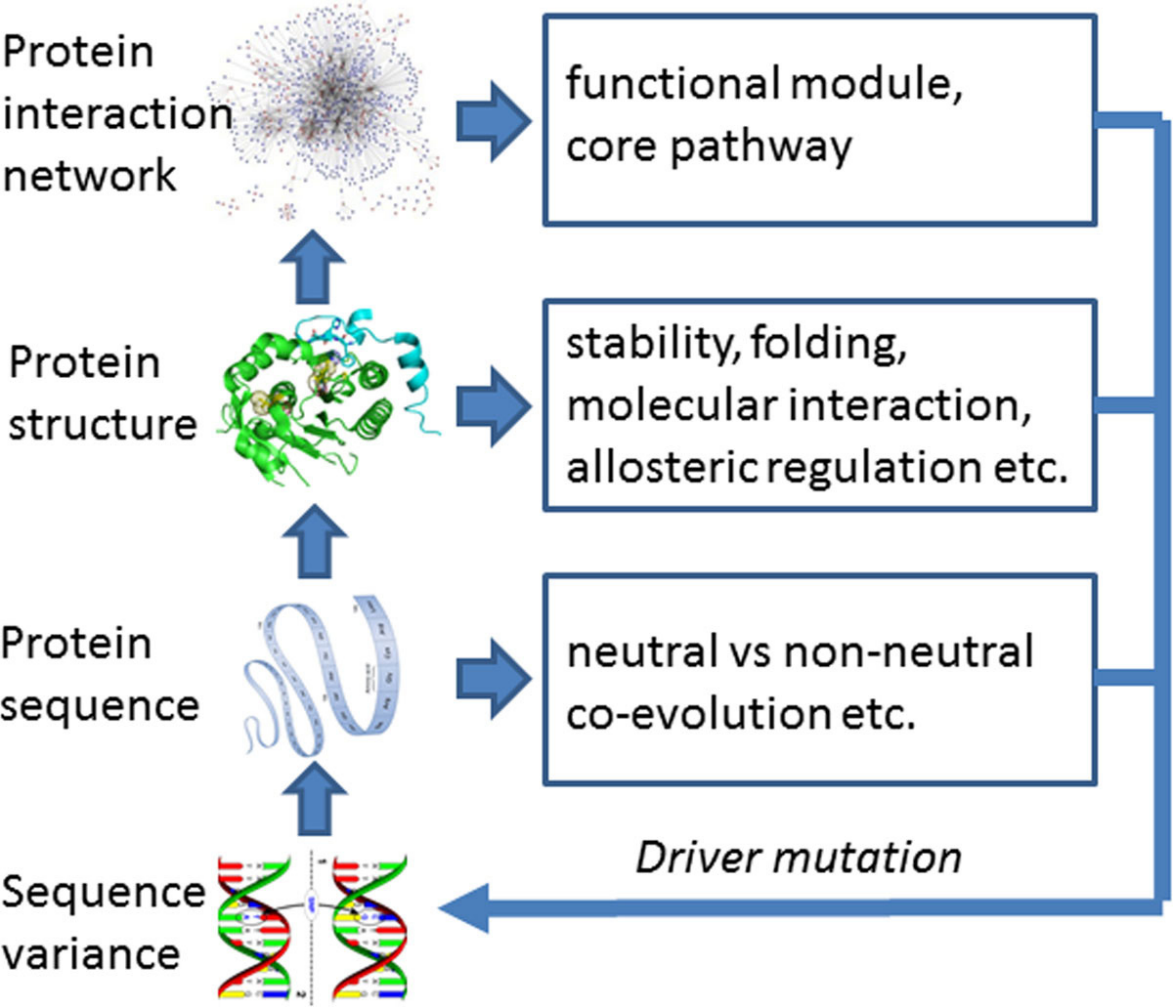
**A multiscale modeling strategy to integrate statistical machine learning, protein structural analysis, and biological network analysis**.

A mechanistic understanding of how individual molecular components work together in a system, and how the system is affected and adapted to individual changes, requires knowledge of molecular structures, their interactions, and their conformational dynamics [19]. Conversely, *a priori *knowledge of structures, their interactions and dynamics may facilitate the identification of causal mutations and their interactions from noisy data even where statistical techniques fail. In this paper, we have developed an integrated multiscale modeling framework to decipher the impact of non-synonymous Single Nucleotide Polymorphisms (nsSNPs) on the information flow from the activity of a single molecular component, to the function of the complete molecular machinery, and ultimately to the emergent properties of the biological network. Conceptually, our approach is rooted in Crick's central dogma of molecular biology and Blois's scalar theory of biomedical information [20]. The fundamental concept of scalar theory is that complex phenotypes arise from the emergent properties of lower scales in the hierarchy which themselves have an intermediate phenotype (or mesophenotype). Based on scalar theory, an organismal phenotype (e.g., disease) emerges from dysregulated pathways that can be identified by genome-wide signatures such as gene expression profiles. The change of the genome-wide signature between disease and normal states results from the altered molecular machinery in the cell, which includes abnormal molecular interactions. In turn, the molecular interaction is determined predominately by the shape, dynamics and physiochemical properties of the associated biomolecules - properties changed by genetic modifications. From an algorithmic point of view, the task is to predict the response of the mesophenotype to the emergent properties of the lower scale, and then use that prediction as input to the upper scale. This is different from current paradigms that often bypass one or more intermediate phenotypes. In practice, each level can be studied independently and then integrated for an improved outcome (Figure 1). In this paper, our contributions are three-fold. First, we address the challenges of identifying causal mutations and epistasis in Genome-Wide Association Studies (GWAS) data when the sample size is extremely small. We do so by incorporating *a priori *knowledge of protein structure, evolution and interaction, and cellular signaling and regulatory pathways. In principle, it allows us to identify driver mutations *de novo*. Second, we show evidence that a large fraction of driver mutations may be involved in perturbation of protein-protein interaction and protein-nucleic acid interactions, and alternation of molecular allosteric regulation; molecular mechanisms that have been paid too little attention in GWAS thus far. Third, we introduce a new method to identify mutation mediated pathway profiles, which can be used to prioritize driver mutations and epistasis, by integrating sequence variances, protein-protein interaction networks, and gene expression profiles.

To demonstrate the feasibility of our approach, we apply multiscale modeling to reveal the genetic, molecular, and cellular basis of hypoxia, a physiological condition in which the cell is deprived of an adequate oxygen supply. The hypoxia-induced phenotype has been related to multiple pathological conditions including cancer [21]. Cells, tissues, and organisms have developed different strategies to survive low oxygen levels; however, the underlying molecular mechanisms contributing to hypoxia tolerance remain unclear. To render mammalian cells and tissues resistant to a low O2 environment, *Drosophila melanogaster *(*D. melanogaster*) has been used as a model system to investigate the mechanisms underlying hypoxia tolerance. Through long-term laboratory selection, Zhou et al. have generated *D. melanogaster *populations that tolerate severe, normally lethal, levels of hypoxia [22]. Microarray analysis identified several adaptive changes in the hypoxia-selected flies [22]. Comparison between the genome sequences of hypoxia-selected flies and those of controls identified 107 amino acid mutations in 52 genes [23]. These data provide us with an unparalleled opportunity to understand the genetic, molecular, and cellular basis of the hypoxia tolerance phenotype and to develop new computational tools to establish causal genotype-phenotype associations, which can be validated through controlled experiments. It is noted that the gene expression profiles are only measured for one condition in the hypoxia tolerance phenotype, hence conventional co-expression approaches are not applicable to this study. Although the hypotheses generated from this study have been experimentally validated by us and are consistent with experimental results from others, the sensitivity and specificity of the method has not been fully evaluated. In the future we will extensively test our method using large case-control datasets from public databases such as the NCBI database of genotypes and phenotypes (dbGap) [24] and the Welcome Trust Case Control Consortium (WTCCC) [25].

## Results

### Knowledge-driven network inference of driver mutations responsible for hypoxia tolerance

**Figure 2:**
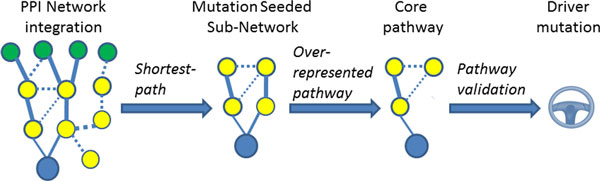
**Workflow to determine core pathways and driver mutations**. (A) mutated genes (blue filled circle) and differentially expressed genes (green filled circle) are mapped to a protein-protein interaction network in which the circles and lines represent proteins and interactions between them, respectively. (B) The shortest-path algorithm is applied to construct subnetworks by linking the mutated genes to up-, or down-regulated genes, respectively. (C) BiNGO is applied to identify overrepresented biological pathways. (D) The experimentally validated driver pathway is used to rank the driver mutations.

Complex phenotypic changes typically arise from re-regulated cellular signaling and regulatory pathways (core pathways). As there are often multiple genes involved in a core pathway, a large number of combinations of genetic alterations can lead to the up- or down-regulation of a pathway. Our hypothesis is that driver mutations will collectively contribute to the re-regulation of a core pathway, which manifests itself as a change to the genome-wide signature, measured here by differentially expressed genes between hypoxia and normoxia phenotypes. In this way it is possible to identify the pathway involved in genotype-phenotype associations from the interacting gene networks that connect the mutated genes to the differentially expressed genes. The pathway that appears more frequently than by chance is a potential core pathway. If the hypothesized core pathway is validated by experiments, or consistent with prior knowledge, the association with the core pathway can prioritize the driver mutation. Based on this rationale, we developed a knowledge-driven network analysis method (Figure 2). First, both mutated genes and differentially expressed genes are labeled in the protein-protein interaction (PPI) network. Second, a mutation seeded subnetwork (MSSN) that connects the mutated gene (seed) and the up- or down-regulated genes (targets) is then identified for each of the mutated genes if the length of the path between the mutated gene and the re-regulated gene is shorter than randomly selected paths. Intuitively, the mutated gene will have a bigger impact on the differentially expressed genes if the distance between them is shorter. Third, the overrepresented biological pathways in the MSSN are identified using BiNGO, a tool for Gene Ontology Over-representation Analysis. The most frequently overrepresented biological pathways for the complete MSSN are hypothesized to be core pathways, and validated by experiment. Finally, the putative driver mutations are ranked by: (1) the statistically significant shortest distance between the mutated gene and the differentially expressed genes in the MSSN, and (2) the statically significant enriched core pathways. This pathway analysis of the MSSN identifies four core pathways: up-regulated Notch and Gurken/Epidermal Growth Factor Receptor (EGFR), and down-regulated Toll and Torso/Receptor Tyrosine Kinase (RTK) pathways. Using Notch signaling inhibition and a P-element screen, we have experimentally validated that the up-regulation of Notch signaling is critical to the survival of hypoxia tolerant Drosophila strains [23,26]. Thus the up-regulation of Notch signaling is confirmed as a driver for hypoxia tolerance in *D. melanogaster*. Although more experiments are needed to validate the direct association of other pathways with hypoxia tolerance and their potential cross-talk with Notch signaling, the mutation could be a driver if it up-regulates Notch signaling. As shown in Table 1, nine MSSNs show statistically significant enrichment (FDR corrected p-value < 0.05) for up-regulation of Notch signaling pathways and significantly shorter paths between the mutated gene and differentially expressed genes. An immediate question is, what are the underlying molecular mechanisms associated with these putative driver mutations? If these mutations are non-neutral at the molecular level, it provides additional support for our hypothesis.

**Table 1:** Predicted driver mutations and core pathways for hypoxia tolerance in *Drosophila melanogaster *from multiple evidences.

Mutated Gene (Annotation Symbol)	Molecular Function	FDR Corrected *p*-value for the overrepresentation of signaling pathways				Shortest-path Distance (z-score) up/down	Functional role of nsSNP inferred from structural modeling	**Expected accuracy (%) of non-neutral mutation from SNAP **[6]	Human ortholog and hypoxia association
						
		Up-regulation		Down-regulation					
						
		Notch*	Gurken/EGFR	Toll	Torso/RTK				
Hairless (CG5460)	transcription corepressor	1.01e-5	2.50e-3	8.23e-3	5.76e-5	2.44/4.42	Possible DNA binding	82	Yes [31]

Rad51D (CG6318)	DNA-dependent ATPase	3.36e-2	1.20e-2	1.95e-2	1.42e-3	2.54/4.09	PPI	<50	Yes [30]

Ulp1 (CG12359)	SUMO-specific protease	4.68e-2	1.87e-2	>0.05	>0.05	1.78/3.86	unknown	63	Yes [43]

Wnt5 (CG6407)	receptor binding	2.67e-2	1.97e-3	1.06e-2	1.16e-7	1.26/3.41	unknown	58	Yes [36-42]

HDAC4 (CG1770)	histone deacetylase 4	2.70e-2	4.10e-4	>0.05	5.76e-5	1.11/3.13	AR of catalytic activity	<50	Yes [34]

Sol (CG1391)	calcium-dependent cysteine-type endopeptidase	1.51e-2	1.69e-2	1.55e-2	2.11e-3	0.33/2.82	unknown	<50	unknown

Dys (CG34157)	Dystrophin	8.28e-5	8.05e-5	>0.05	3.17e-3	0.38/0.72	AR of substrate binding	70	Yes [32,33]

GalNAc-T2 (CG6394)	N-acetylgalactosaminyl transferase	2.59e-3	2.71e-10	1.30e-2	3.28e-3	-1.51/0.81	AR of substrate binding	<50	Yes [35]

CG33714 (CG33714)	mRNA binding	8.59e-5	5.61e-3	1.51e-2	>0.05	-1.59/0.69	mRNA binding	87	unknown

*We experimentally validate that the up-regulation of Notch signaling, one of the most frequently overrepresented pathways, confers the hypoxia tolerance in *Drosophila melanogaster *[23, 26]. PPI: Protein-Protein Interaction, AR: Allosteric Regulation.

### Structural analysis of functional roles of nsSNPs

#### Structural modeling of nsSNPs

**Figure 3:**
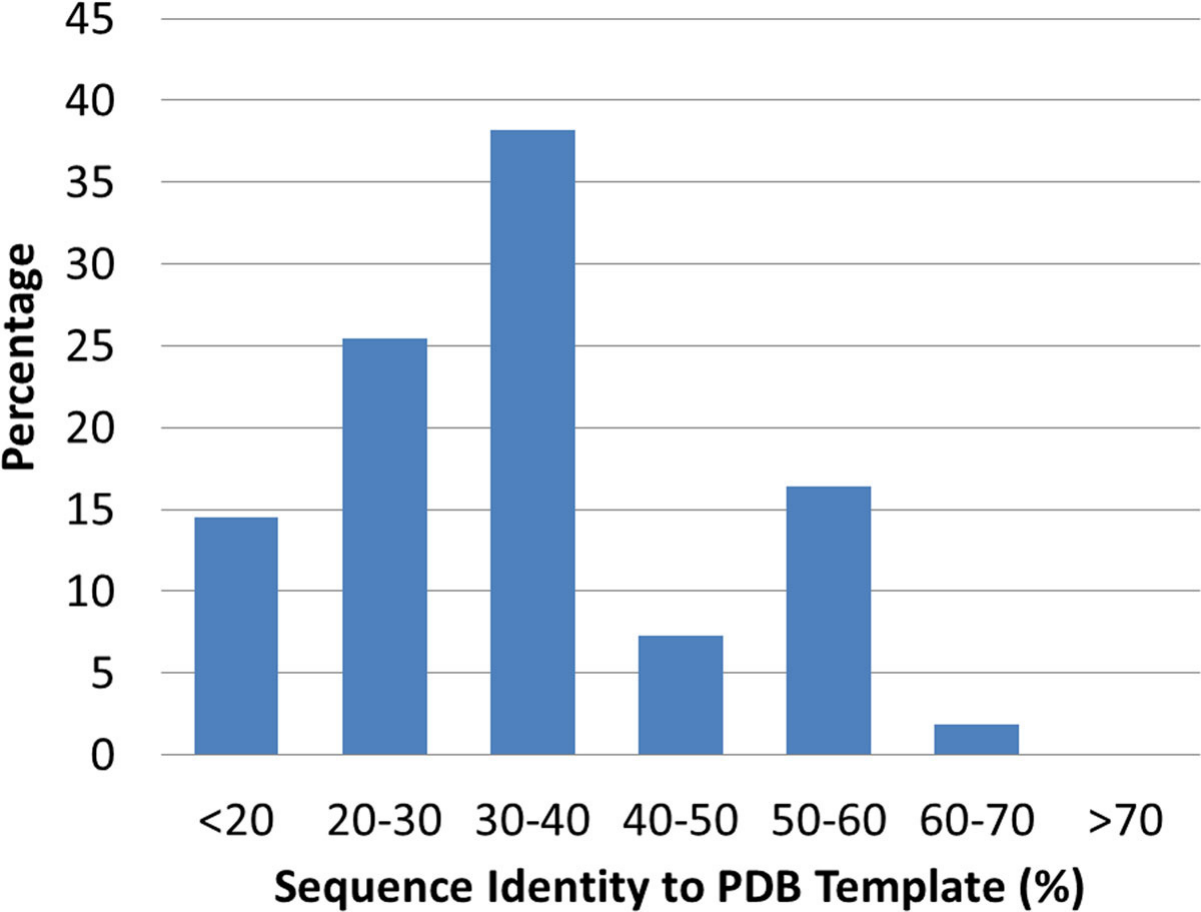
**Distribution of sequence identities between proteins containing nsSNPs and templates in the RCSB PDB**.

To better understand the molecular basis of potential driver mutations, we mapped point mutations to protein structure models. Among the 52 proteins containing nsSNPs, none of them have known structures available in the RCSB Protein Data Bank (PDB) [27]. Homology models were built for these proteins. The distribution of the sequence identities associated with the structural templates used in modeling is shown in Figure 3. 60% of models are based on a template with a sequence identity greater than 30%, a common threshold for building reliable homology models. These protein models can be grouped into four categories: 1) Reliable models can be built and the locations of mutations are close to known functional sites. Thus, the functional role of nsSNPs can be predicted in a relatively straightforward manner. These proteins (6 total) are listed in Additional File 1 table S1 and model structures of these proteins are shown in Additional File 1 Figure S1. 2) Reliable models can be built but the point mutation cannot be mapped to any functional sites. This category includes 11 proteins (Table S2). 3) No structural templates can be found for the domains containing the mutation, but structural models can be built for other functional domains of the same protein. 21 proteins are in this category (Table S3). 4) No structural templates can be found for the whole protein or any part; 15 proteins are in this category.

#### Structural roles of putative driver mutations

**Figure 4:**
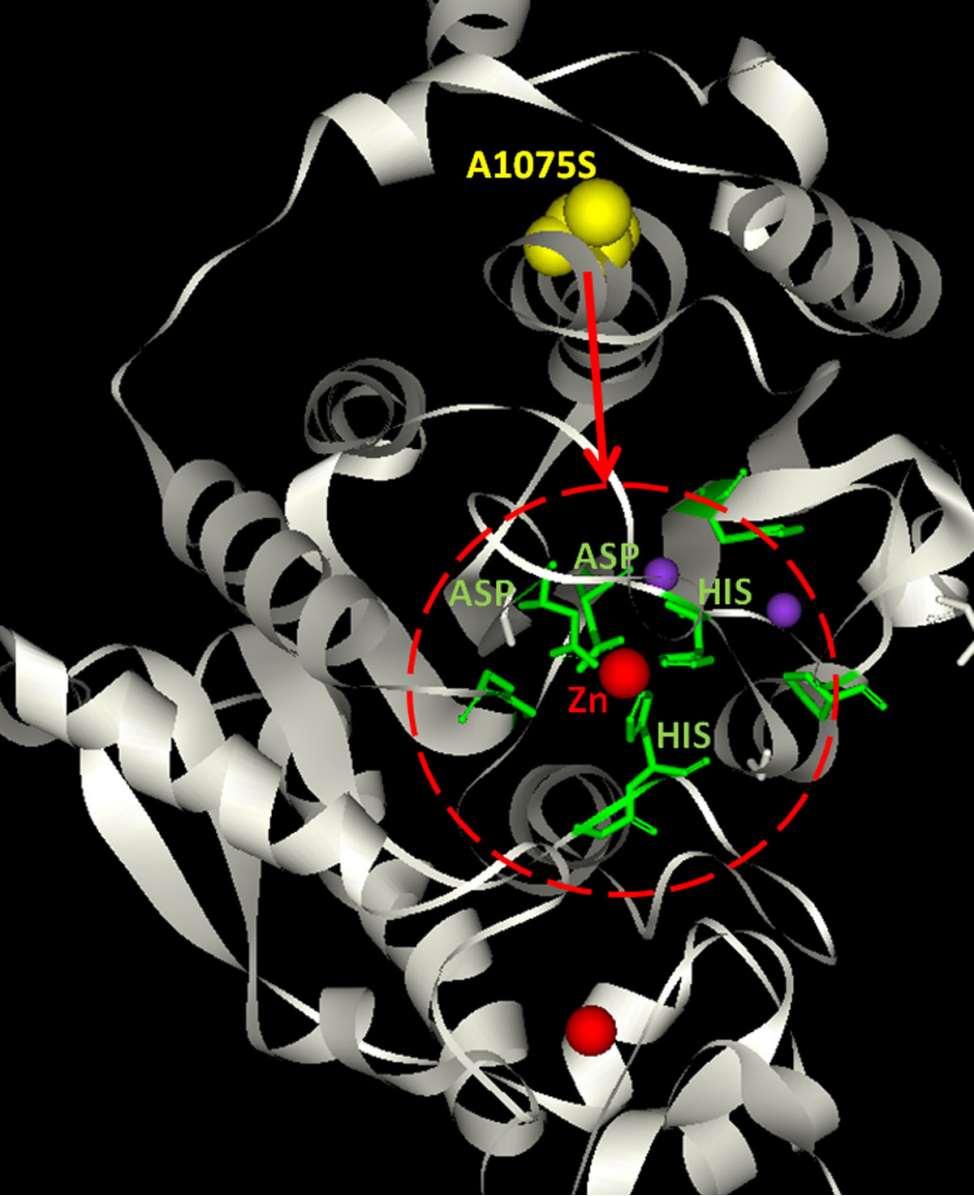
**The model structure of HDAC4**. The yellow spheres represent mutated amino acid A1075. Green sticks represent residues co-evolved with A1075. Red circle represents zinc binding sites of HDAC4. This model structure is built using Modeller [65] based on the sequence alignment between HDAC4 and PDB structure 2VQW. The sequence identity between HDAC4 (819-1223) and 2VQW is 58%.

For putative mutations in nine genes predicted from network analysis (Table 1), we first analyze if the mutation might alter substrate binding, catalytic activity, or structural stability. Interestingly, most of the predicted driver mutations are surface-exposed, but not located in conserved functional sites. We hypothesize that they may be involved in allosteric regulation, protein-protein interactions, or protein-nucleic acid recognition. Co-evolution analysis is applied to these proteins to identify the correlation between mutated amino acids and functional sites. The residue couplings were observed in four structures (HDAC4, Dys, GalNAc-T2, and CG33714). One example is HDAC4, which belongs to the histone deacetylase family. As shown in Figure 4, residues that are predicted to be co-evolved with A1075, one of the mutations in HDAC4, form zinc binding sites. Among them, the two His residues around the zinc ion are conserved in all members of the class IIa histone deacetylase family. The mutation of residues coordinating the zinc ion was reported to prevent the association of HDAC4 with the N-CoR· HDAC3 repressor complex [28], which is required for HDAC4 to possess histone deacetylase activity [29]. Thus, A1075 is functionally coupled to the zinc binding site in HDAC4, and as a consequence, may remotely regulate its activity. More examples are shown in the Additional File 1 Figures S2-S4.

**Figure 5:**
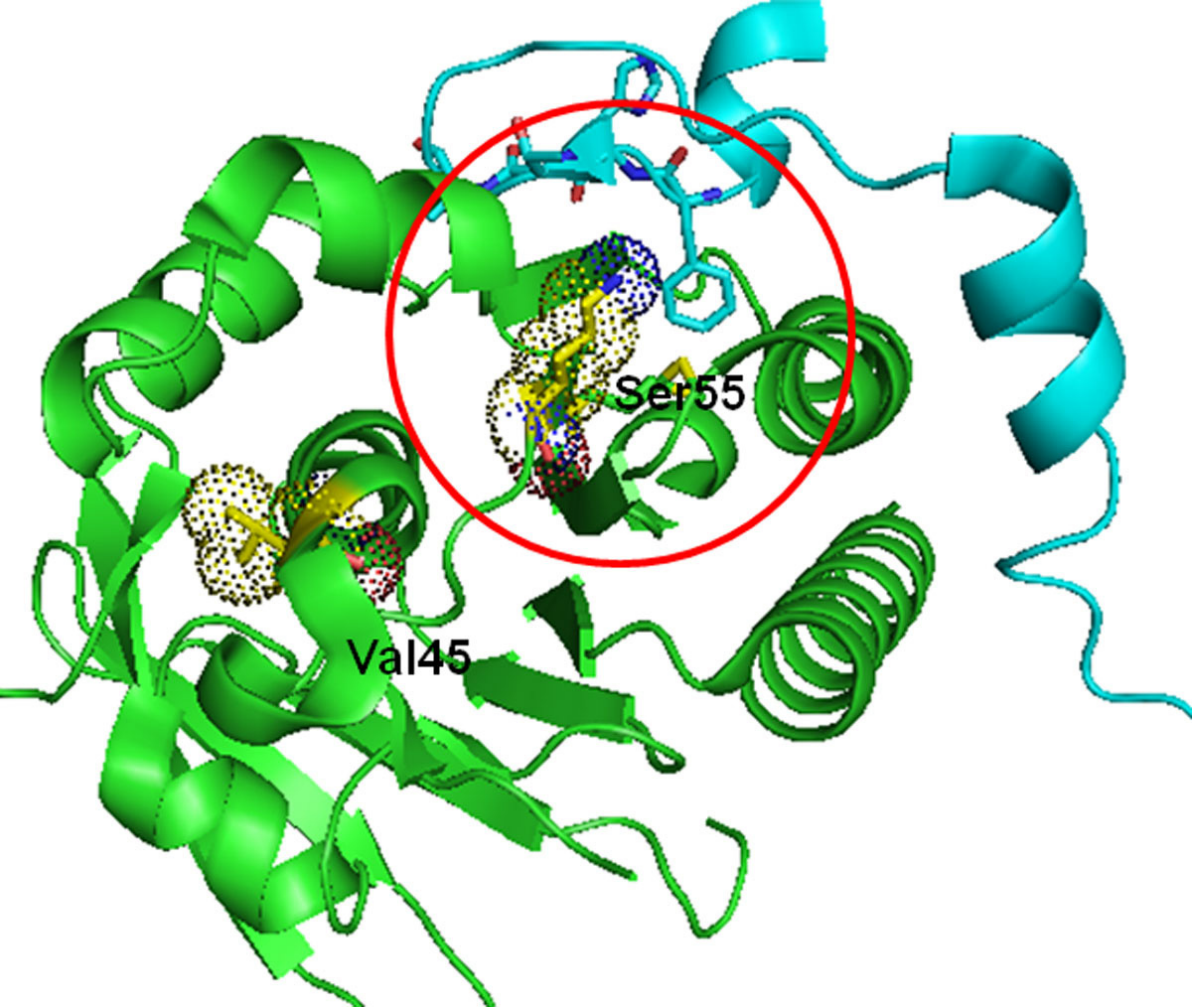
**Model structure for Rad51D**. The green and cyan cartoon represents the model structure of Rad51D and BRCA2, respectively. Dotted spheres represent the mutated amino acids. The red circle indicates the region of the protein-protein interaction interface. Ser55 mutation may directly impact the oligomerization of Rad51D.

In addition to allosteric regulation, the putative driver mutation may modify protein-protein interactions. This is the case for Rad51D, as shown in Figure 5. Rad51D plays a major role in homologous recombination repair (HRR) of damaged DNA arising during replication or induced by DNA damaging agents. BRC repeat (BRCA2 in Figure 5) mimics a motif in Rad51D that serves as an interface for oligomerization between individual Rad51D monomers. One of the Rad51D mutations, S55N, is close to the oligomerization interface between individual Rad51D monomers and hence may impact the formation of the Rad51D complex, which is associated with the hypoxia phenotype [30].

### Machine learning based prediction of non-neutral nsSNPs

The functional importance of nsSNP is further supported by SNAP [6], software used to predict a given nsSNP as neutral or non-neutral with an expected accuracy. In a benchmark study, SNAP outperformed most similar methods [6]. 23 out of the 107 nsSNPs, located on 18 genes, are predicted as non-neutral with an accuracy of higher than 58% (SNAP reliability index 0), (Additional File 1 Table S4). Five predicted non-neutral mutations are hypothesized as putative drivers. Two of them (H and CG33714) have an accuracy of over 80%. The remaining predictions have lower expected accuracies. This could imply that while the functional impact of each individual mutation is limited, collectively they may mediate the signaling pathway activity through epistasis.

Several mutations in CG31220 (Additional File 1 Table S4), a serine-type peptidase, are predicted as non-neutral by SNAP. These mutations are mapped to the substrate binding sites or other functional important regions in the structure (Additional File 1 Figure S1). However, enriched biological pathways associated with this gene were not detected. More studies are required to understand how these non-neutral mutations impact the biological network.

### Experimental and literature supports

As discussed above, a complex phenotype rises from re-regulated biological pathways that themselves result from the collective effects of multiple genetic mutations (epistasis). Since the down- or up-regulation of core pathways directly impacts the organismal phenotype, the experimental validation of the core pathway would provide strong evidence to support the predicted driver mutations that are responsible for the re-regulation of the core pathway. Indeed, we have experimentally validated that Notch signaling is the core pathway of hypoxia tolerance in *D. melanogaster*. The reduced activation of Notch signaling by a specific γ-secretase inhibitor significantly reduces the survival and life-span of hypoxia tolerant *D. melanogaster *strains [23]. The critical role of Notch signaling in hypoxia tolerance is further supported by UAS-Gal4 over-expression and RNAi knockdown of genes involved in Notch signaling [26]. Other experimental evidence from the literatures, as detailed below, also support our predictions. The top ranked H gene (also called hairless) is a well-known regulator of Notch signaling in *D. melanogaster *[31]. Dys encodes the protein dystrophin. Genetic interaction screens in *D. melanogaster *have shown that Dys is involved in interactions with components of the Notch signaling pathway [32]. Furthermore, the mutation of the Dys homolog in the mouse model is related to the up-regulation of the Notch-beta pathway [33]. For other genes, although little direct experimental evidence supports an association with hypoxia in *D. melanogasta *their functional roles in hypoxia has been demonstrated in cancer and other human diseases. HDAC4 regulates hypoxia-inducible factor 1 α (HIF1 α) and cancer cell response to hypoxia [34]. GalNAc-T2 is an N-acetyl-galactoseaminyl transferase that catalyzes the synthesis of glycosphingolipid (GSL). A recent study has shown that GSL may directly regulate the activity of Notch signaling [35]. Wnt5 is a ligand to a family of frizzled receptors, acting as a regulator of Wnt signaling. An increasing body of evidences suggests that Wnt and Notch signaling cooperatively determine the fate of cell development in humans [36-42]. The association between Rad51D and hypoxia has been demonstrated in cancer [30]. Ulp1 is a SUMO-specific protease that is essential for the stabilization of HIF1α during hypoxia by removing SUMO and participates in the regulation of hypoxia-responsive genes [43].

## Discussion

### The important functional role of allosteric regulation, protein-protein interactions, and protein-nucleic acid interactions in sequence variants

In this study, none of the driver mutations associated with hypoxia are conserved functional site residues, nor are they responsible for structural stability. The driver mutations are hypothesized to be involved in either protein-protein interactions (in the case of Rad51D), protein-nucleic acid interaction (e.g., in CG33714), or allosteric regulation (e.g., in HDAC4). A recent survey of the structural basis of in-frame mutations in protein-protein interactions has suggested that changes in specific interactions play a critical role in pathogenesis [44]. From a network point of view, the modification of protein-protein interactions, rather than the proteins themselves, may have significant impact on network properties [45]. Recent progress in the ENCODE and modENCODE projects highlights the critical functional roles of non-coding DNAs in the regulation of biological processes [46,47]. As a large number of non-coding DNAs perform their functions through specific protein-nucleic acid interactions, the mutations that impact protein-nucleic acid binding could be directly associated with phenotype changes. The dysregulation of allosteric interactions is considered to be another major determinant of disease [48]. During evolution, organisms need to survive and reproduce in a changed environment. As such, certain genes need to gain functions and activate critical pathways. Allosteric regulation is an efficient way for driver mutations to act since the change of activity is not constrained to a single molecule, but can be propagated to a whole network [19]. New computational methods that are able to identify "hot spots" in protein-protein interactions, protein-nucleic acid recognition, and allosteric regulations, in which the mutation may cause the dysregulation of biological pathways, may have significant impact on the interpretation of Genome-Wide Association Studies.

### The relevance of *D. melanogasta *driver mutations to human hypoxia adaption

Recently several studies in hypoxia adaptation in humans have been performed on Tibetans [49,50], Andeans [50], and Ethiopians [51]. However, all human studies to date have adopted limited, sampling-based approaches, such as genotyping or exome sequencing. The relatively sparse sampling of the genome makes it harder to identify large-scale shifts in the allele frequency spectrum associated with natural selection. Consequently, these studies restricted subsequent analysis to variants in candidate genes that are mainly involved in the canonical hypoxia response (HIF pathway) and related pathways. The identification of the functional roles of sequence variances in human orthologs of *Drosophila *genes may provide critical insight in the prioritization of candidate genes in human, which may fail using conventional statistical techniques. Indeed, the majority of driver mutations identified in this study are human orthologs and associated with the hypoxia cellular phenotype, as shown in Table 1.

## Conclusion

Based upon multiscale modeling, we propose that the up-regulation of Notch and Gurken/EGFR and the down-regulation of Toll and Torso/RTK pathways are responsible for hypoxia tolerance. Using integrated structural and network analysis, we hypothesize that nsSNPs in H, Rad51D, Ulp1, Sol, Wnt5, CG33714, GalNAc-T2, Dys, and HDAC4, may all lead to the functional modification of these genes via allosteric regulation and protein-protein/DNA/RNA interactions and hence are driver mutations defining the hypoxia tolerance phenotype. Our predictions are supported by experimental evidence [23,26]. Moreover, multiscale modeling may identify potential epistasis using a very small sample size. This reduces the burden imposed during statistical multiple testing of large epistasis models. It is anticipated that the further extension of this multiscale modeling approach to genome-wide protein-protein interactions, protein-nucleic acid interactions, and microRNA data will provide a powerful tool for uncovering the functional roles of both coding and non-coding sequence variations in GWAS; a role which neither the knowledge of molecular structures nor of biological networks alone can achieve. However, challenges remain in extending multiscale modeling approaches. New algorithms are required to predict emergent properties, at both molecular and network levels, as well as to seamlessly model information flow across scales.

## Methods

### Prediction of non-neutral mutations on nsSNPs from sequence

A sequence information based method, SNAP [6] is used to predict the non-neutral (functional effect) and neutral (no functional effect) nsSNPs.

### Knowledge-driven network inference of core pathways and driver mutations

#### Overview

The network-based analysis of driver mutation is shown in Figure 2. The mutated genes and differentially regulated genes are mapped to a protein-protein interaction (PPI) network extracted from the STRING Database [52] for *D. melanogaster*. A subnetwork that connects a mutated gene and up-, and down-regulated genes is identified using a shortest path search of the PPI network. The genes identified in each subnetwork are subject to Gene Set Enrichment Analysis (GSEA). If the genes in the subnetwork are enriched by the essential biological processes/pathways, the mutated gene is a potential driver.

#### Analysis of differential expressed genes

A cDNA microarray analysis of 13,061 known or predicted genes from the *D. melanogaster *genome is performed using the R package [53]. K-nearest neighbors [54] in the space of genes is used to impute missing expression values. The LOWESS normalization method [55] is used to normalize the raw density data. P-value and fold change are calculated using the two-sided, two-class t-test [56]. A Bonferroni-Holm [57] false discovery rate (FDR) controlling procedure [58,59] is used to adjust the P-values. The genes are considered to be differentially expressed between the two samples when the FDR is smaller than 0.05. If the fold change is larger than 1.5-fold for up-regulated genes and is smaller than 0.67-fold for down-regulated genes, these genes are considered significantly differentially expressed.

#### Subnetwork construction by shortest path search

A program based on Dijkstra's algorithm [60] is developed to search for the shortest path from a source node (mutated gene) to a destination node (differentially expressed gene) in a protein-protein interaction (PPI) network. A shorter path implies that the mutated gene has stronger influence on the differentially expressed genes. All genes along the path form a subnetwork. In order to obtain a quantitative measurement to distinguish the different topologies in the subnetworks, a t-value based on Welch's t-test is calculated for each mutated gene. The Welch's t-test [61] calculates the difference of two populations whose variances are assumed to be different (unequal sample size and unequal variance). The t-value is calculated as follows:

$$t=\frac{{\bar{x}}_{1}-{\bar{x}}_{2}}{\sqrt{\frac{s_{1}^{2}}{n_{1}}+}\frac{s_{2}^{2}}{n_{2}}}$$

Where *s*^2 ^is the unbiased estimator of the variance of the sample and n is the number of participants.

Here the t-value is used to measure the difference between the identified subnetwork (x_2_) and a background random network (x_1_). Background random networks are built by randomly selecting one gene as a source node and a set of other genes as destination nodes. A positive t-value means a shorter than average path. The mutations on the genes with statistically significant high t-values are prioritized as driver mutations.

#### Gene set overrepresentation analysis to identify driver biological pathways and mutations

The Biological Networks Gene Ontology Tool (BiNGO) [62] is applied in Cytoscape's versatile visualization environment [63] to determine which biological processes and molecular functions are significantly overrepresented in the set of genes involved in each subnetwork. Gene ontology [64] terms are ranked according to the False Discovery Rate (FDR) corrected p-values for each subnetwork. The statistically significant enriched biological pathways (p-value < 0.05) are considered as potential core pathways that contribute to the survival or reproduction of a phenotype. This pathway is subject to further validations by experiments and literature searches. If a subnetwork contains the validated core pathway, the mutated gene in this subnetwork is hypothesized to be a causal gene. Correspondingly, the mutations on this gene are candidate driver mutations.

### Structure-based analysis of driver mutations

#### Homology modeling and nsSNP mapping

Homology models of proteins are built using Modeller [65]. Sequence alignments between these proteins and templates of known structures are obtained from a PSI-BLAST sequence search [66]. The functional sites are predicted using *SMAP *[67-69]. Mutated residues are mapped onto the model structures and the functional roles of these residues are predicted according to their locations on the model structures.

#### Covariance analysis

Covariance analysis based on multiple sequence alignments of proteins in the same Pfam family [70] as the mutated protein can help identify remote relationships between mutated residues and other residues within the protein sequence. The Pfam family is identified by a whole sequence search. Redundancy of sequences in the Pfam family is removed using CD-hit [71] with a sequence identity threshold of 90% [72]. Multiple sequence alignments among these sequences are built using the MUSCLE software [73] with default parameters. Covariance of mutations with other residues is calculated using five different methods: Statistical Coupling Analysis (SCA) [74]; Explicit Likelihood of Subset Co-variation (ELSC) [75]; Observed Minus Expected Squared covariance algorithm (OMES) [76]; Mutual Information Covariance Algorithm (MI) [77]; and Conservation Algorithm (ConservationSum) [78]. The residues that are predicted to be coupling with mutations by at least two methods are considered as co-evolved residues with the mutated residues.

## Availability of supporting data

The data sets supporting the results of this article are included within the article.

## List of abbreviations

Genome-Wide Association Studies (GWAS), Epidermal Growth Factor Receptor (EGFR), Receptor Tyrosine Kinase (RTK), Mutation Seeded Subnetwork (MSSN), Protein-Protein Interaction (PPI).

## Competing interests

Authors declare that they have no competing interests.

## Authors' contributions

Conceived and designed the experiments: Lei Xie. Performed the experiments: Li Xie, Dan Zhou, Clara Ng, Thahmina Ali, Raoul Valencia, Barbara L. Ferreira, Vincent Xue, Maliha Tanweer, Analyzed the data: Li Xie, Lei Xie. Contributed reagents/materials/analysis tools: Li Xie. Dan Zhou, Gabriel G. Haddad, Wrote the paper: Li Xie, Lei Xie, Clara Ng, Philip E. Bourne.

## Supplementary Material

Additional file 1 Figure S1. Structure models for proteins with mutations that are close to substrate binding sites or active sites.
Figure S2. The structural model of CG33714.
Figure S3. The structure model of GalNac-T2.
Figure S4. The structural model of WW domain of Dys.
Table S1 Genes in the first category.
Table S2. Genes in the second category.
Table S3. Genes in the third category.
Table S4. Non-neutral mutations predicted by SNAP. Click here for file

## Declarations

The publication costs for this article were funded by CUNY Research foundation.

This article has been published as part of *BMC Genomics *Volume 14 Supplement 3, 2013: SNP-SIG 2012: Identification and annotation of SNPs in the context of structure, function, and disease. The full contents of the supplement are available online at http://www.biomedcentral.com/bmcgenomics/supplements/14/S3
