# Supplementary material for: Multiscale modeling of the causal functional roles of nsSNPs in a genome-wide association study: application to hypoxia
Source: BMC Genomics. 2013 May 28;14(Suppl 3):S9. doi: 10.1186/1471-2164-14-S3-S9 (PMC3665574; doi:10.1186/1471-2164-14-S3-S9)
Supplement: Additional file 1 — Figure S1. Structure models for proteins with mutations that are close to substrate binding sites or active sites. Figure S2. The structural model of CG33714. Figure S3. The structure model of GalNac-T2. Figure S4. The structural model of WW domain of Dys. Table S1 Genes in the first category. Table S2. Genes in the second category. Table S3. Genes in the third category. Table S4. Non-neutral mutations predicted by SNAP. [file 1471-2164-14-S3-S9-S1.PDF]

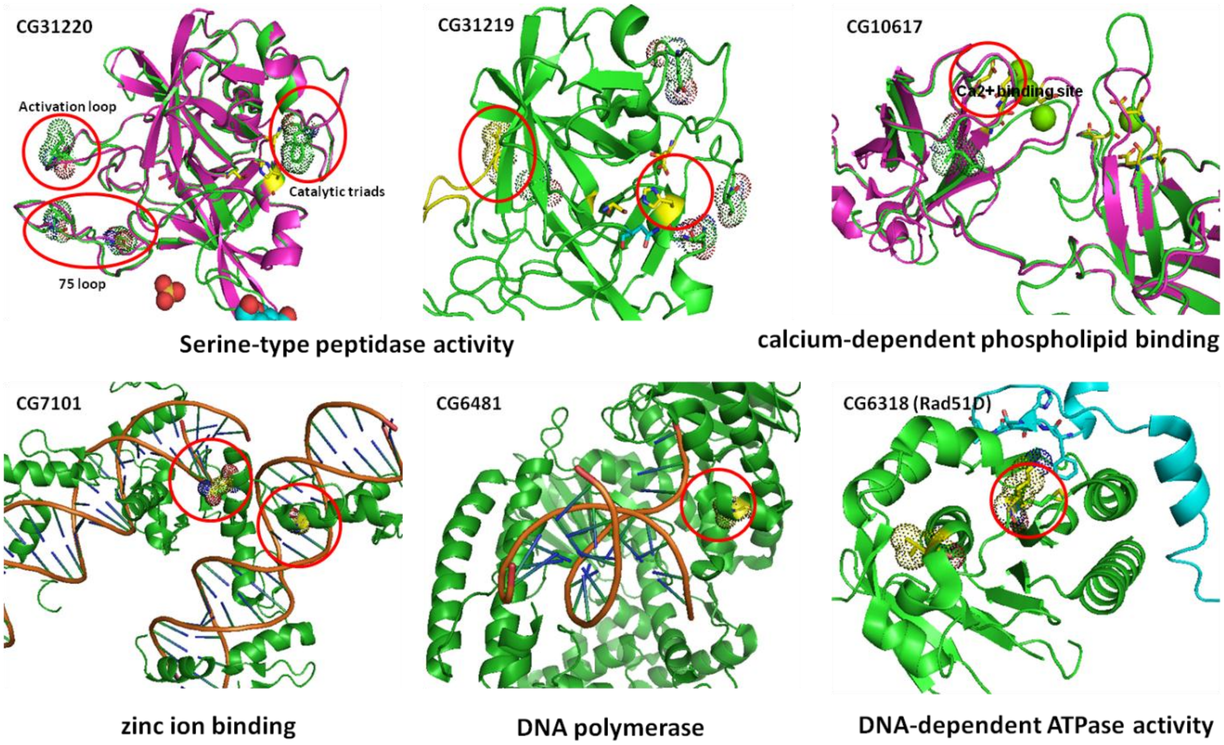


**Figure S1. Structure models for proteins with mutations that are close to substrate binding sites or active sites.** The molecular function of CG31220 is characterized as having serine-type peptidase activity. The best structural template for this protein is a serine protease domain of prophenoloxidase activating factor-I in a zymogen form (PDB id: 2OLG, 37% sequence identity). Mutations on this protein are I42M, V124I, T128I and F150S. I41 and F150 locate on the activation loop, and near catalytic triads, respectively. V124 and T128 are on the 75-loop, the function of which is to prevent spontaneous activation of the peptidase [1]. Moreover, V124 and T128 are close to a conserved S-S disulphide bond on 75-loop. The mutations may contribute to maintaining the S-S bond under a low oxygen concentration. CG21220 is not predicted as a driver. The implications of this mutation on hypoxia adoption remain unclear. SNAP does not predict mutations on four other proteins as being non-neutral. However, several of these mutations may affect the activity of the proteins. For example, CG31219 is a serine-type endopeptidase. The mutations that are associated with hypoxia tolerance include Q82R, N138D, S193N, A237V and Q275K. Q82 is on the activation loop and close to the S-S bond; N138 is on the 60-loop and close to the catalytic triads; and A237 is close to the 140-loop. Another protein Syt12 is predicted to be involved in calcium-dependent phospholipid binding. One of mutations I514M is close to the Ca2+ binding site. The function of CG6481 and CG7101 are predicted to be a DNA polymerase and zinc binding protein, respectively. The mutations on these proteins may affect nucleic-acid binding. Although more detailed energetic and dynamics analysis is required to reveal the physical origin of these mutations, the structural analysis provides critical clues to their functional roles, and identifies potential non-neutral mutations missed by SNAP.

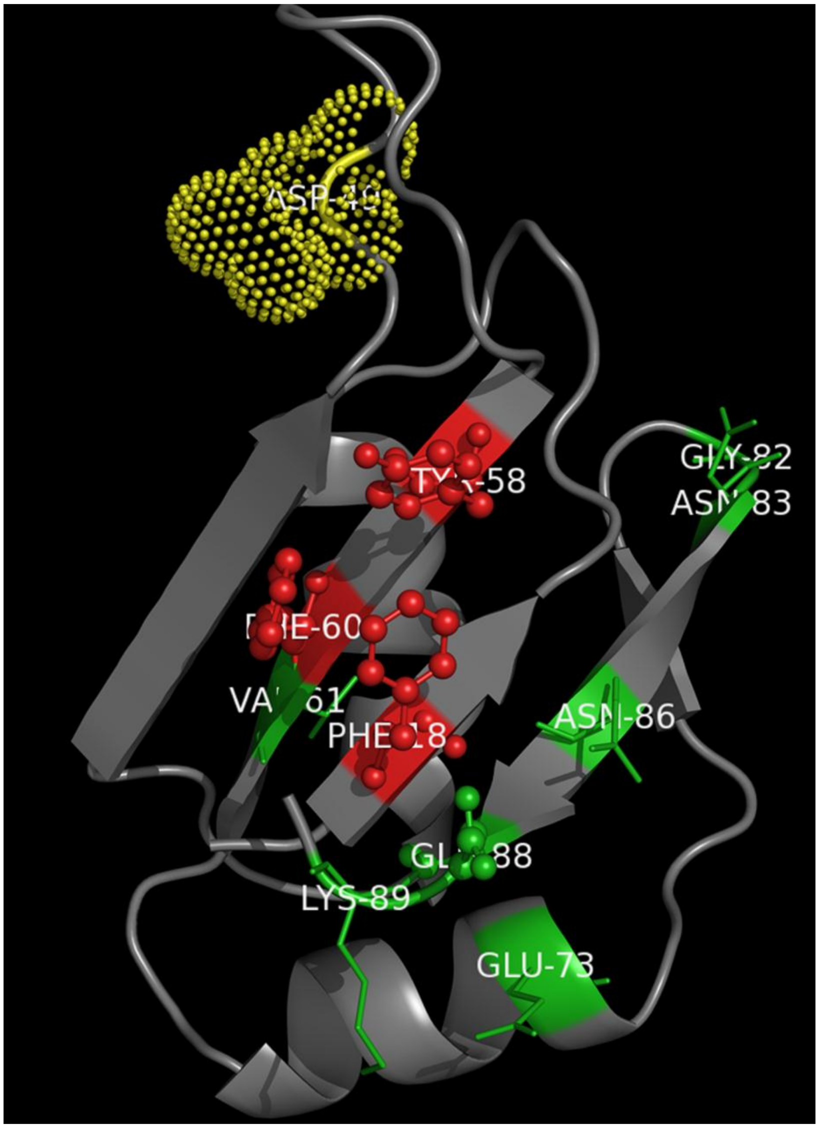


**Figure S2. The structural model of CG33714.** The yellow dotted spheres represent the mutated residue D49. Red sticks and balls represent the predicted active sites, F18, Y58 and F60. Green sticks represent the residues co-evolved with D49, including V61, E73, G83, N83, N86 and K89. Green ball and sticks represent N88, which is predicted as an active site and co-evolved residue. Co-variance analysis was also applied to CG33714, a nucleotide-binding protein. The structural mode was built by Modeller [2] based on the sequence alignment between CG33714 and PDB structure 1HD0. The sequence identity is 32%. Among co-evolved residues, V61 is close to Y58, F60 and F18, which were predicted to form stacking interaction with RNA bases according to the template structure. N86, Q88 and L89 are located on the same strand and which is anti-parallel to the strand containing RNA binding residue F18. Q88 is predicted to form a hydrogen bond interaction with the RNA target. Thus, the D49A mutation in CG33714 may allosterically regulate RNA binding.

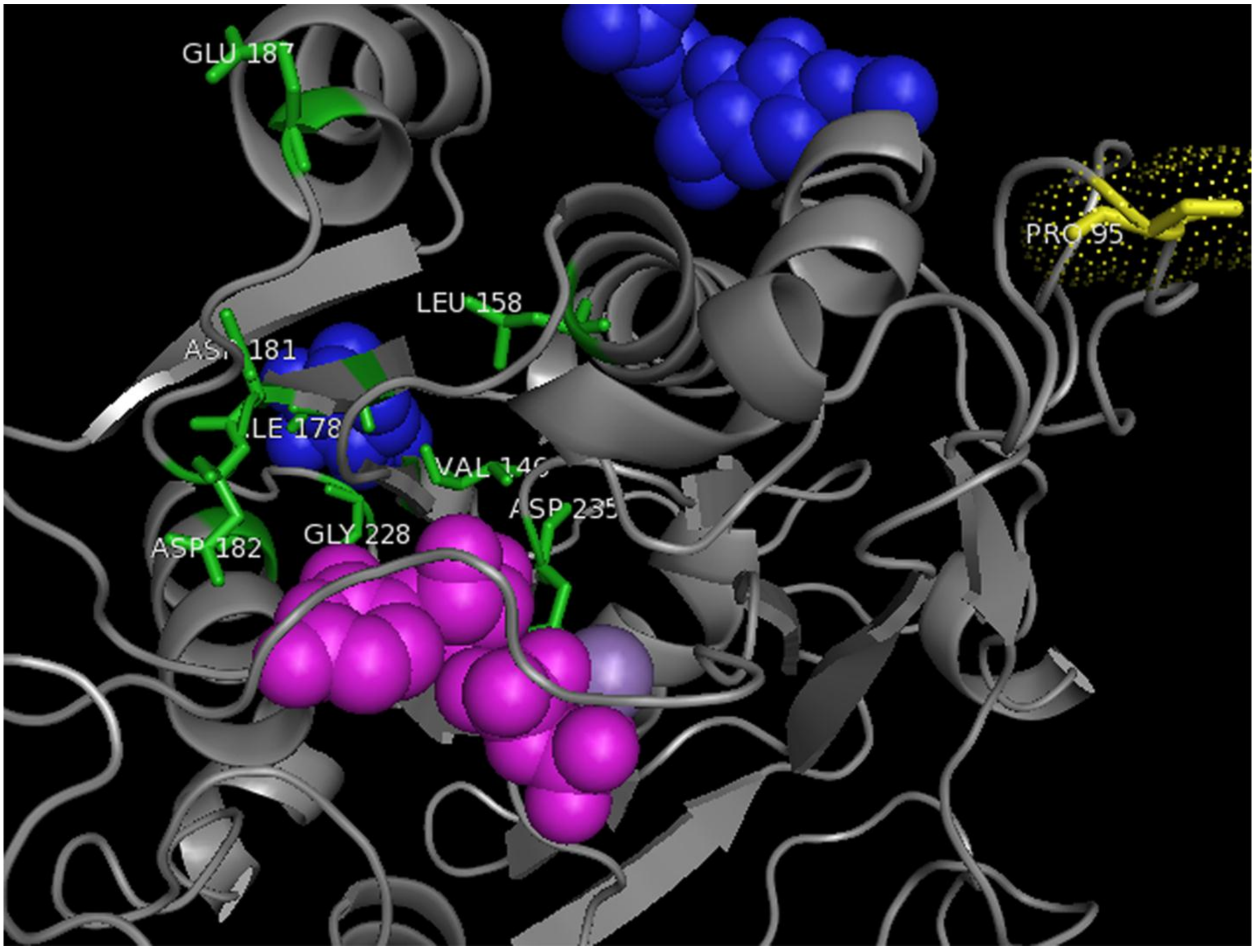


**Figure S3. The structure model of GalNac-T2.** Yellow dotted spheres represent the mutated residue P95. Green sticks represent residues co-evolved with P95, including V146, L158, I178, D181, D182, G228 and D235. Magenta spheres represent the substrate UDP and blue spheres represent the substrate. Through co-evolution analysis, the mutation on GalNac-T2 (P95) was found to be related to the ligand binding site. The model was built based on the sequence alignment between GalNac-T2 and a human GalNac-T10 (PDB ID: 2D7R). The sequence identity between them is 41%. 2D7R is co-crystallized with the hydrolyzed donor substrate uridine-5'-diphosphate (UDP) and N-acetyl-D-glucosamine (NAG) [3]. The conformations of UDP-GalNac and GalNac-ser are superimposed on the model structure and indicate where the potential binding pockets are located for the *D. melanogaster* GalNac-T2. Two co-evolved residues, D182 and D235 interact with the substrate UDP and the other two, I178 and G228 interact with NAG. Other co-evolved residues such as V146, L158 and D181, are close to the binding pockets even though they do not directly interact with these substrates. The interactions between the co-evolved residues and substrates potentially indicate remote regulation of P95 during the enzyme reaction.

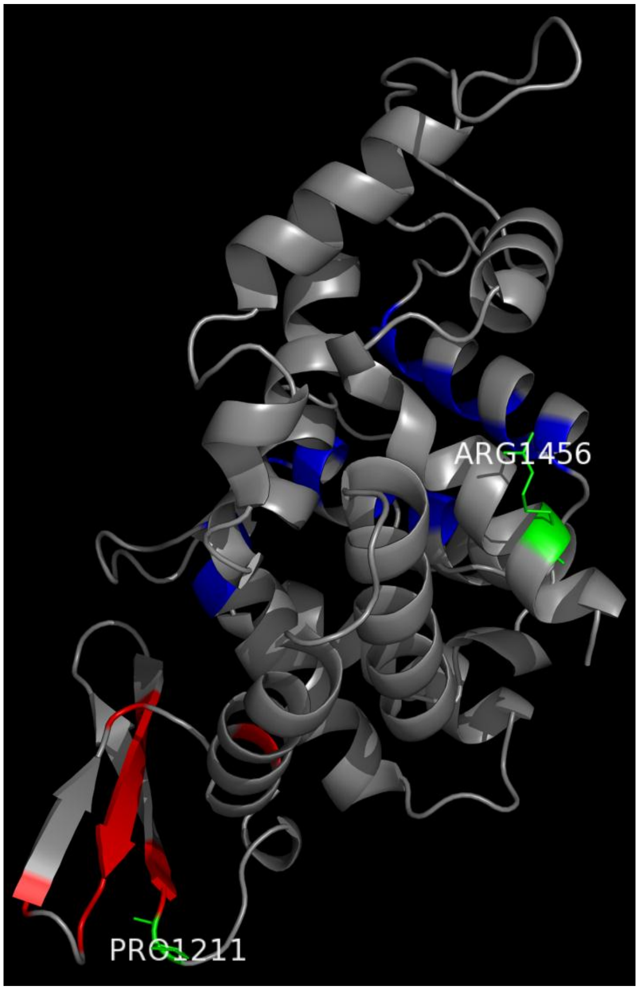


**Figure S4. The structural model of WW domain of Dys.** Green sticks represent the residues co-evolved with L24. Red represents the predicted binding site of the β-dystroglycan peptide. Blue represents the interface between the two EF-hands. Co-evolved residues were predicted associated with the mutated L24 on dystrophin (Dys). Unlike the mutations on HDAC4 and CG33714, reliable structural templates to build model structures are absent for the domain containing L24, thus a structural view is not available. However, the structural model for other domains of Dys can be built using homology modeling. Co-evolved residues found on these other domains could be mapped to the model. The structural model of WW domain of Dys was built based on the structure of 1EG3, a human dystrophin WW domain. The sequence identity between them is 57%. Dystrophin interacts with β-dystroglycan to form a dystrophin-glycoprotein complex that spans the cell membrane and links the actin cytoskeleton to the extracellular lamina [4]. The structure of the WW domain contains two EF-hand-like motifs. The β-dystroglycan peptide binds to the surface formed by the WW domain and one EF-hand motif. The residues that interact with β-dystroglycan are shown in red in the figure. P1211, one of the co-evolved residues of the L24 mutation is next to this interface. It is speculated that the L24Q mutation can alter the activity of Dys through allosteric regulations. Another co-evolved residue, R1456 is close to the interface between the two EF-hands.

**Table S1 Genes in the first category.**

| Gene Symbol | Molecular function | Biological process | Template sequence identity |
|---|---|---|---|
| CG31220 | Serine-type peptidase activity | proteolysis | 37% |
| CG31219 | serine-type endopeptidase activity | proteolysis | 35% |
| Syt12 | calcium-dependent phospholipid binding | neurotransmitter secretion | 33% |
| Rad51D | DNA-dependent ATPase activity | DNA recombination DNA repair | 21% |
| CG6481 | DNA Polymerase | - | 27% |
| CG7101 | zinc ion binding | - | 30% |

**Table S2. Genes in the second category.**

| Gene Symbol | Molecular function | Biological process | Template sequence identity |
|---|---|---|---|
| IP3K2 | calcium-dependent protein binding | inositol and derivative phosphorylation | 61% |
| HDAC4 | histone deacetylase activity | regulation of transcription | 58% |
| GalNAc-T2 | polypeptide N-acetylgalactosaminyltransferase activity | oligosaccharide biosynthetic process | 41% |
| CG1764 | dimethylargininase activity | - | 38% |
| CG6470 | zinc ion binding | | 38% |
| CG33714 | mRNA binding | - | 32% |
| CG6461 | gamma-glutamyltransferase activity | - | 30% |
| Aats-his | histidine-tRNA ligase activity | histidyl-tRNA aminoacylation | 27% |
| CG9981 | ATPase activity | ATP biosynthetic process | 27% |
| CG7342 | secondary active organic cation transmembrane transporter activity | - | 25% |
| CG1486 | pyridoxal phosphate binding; carboxy-lyase activity | carboxylic acid metabolic process | 24% |

**Table S3. Genes in the third category.**

| Gene Symbol | Molecular function | Biological process | Template Sequence identity |
|---|---|---|---|
| Dys | WW domain binding | neuromuscular synaptic transmission | 59% |
| HDAC4 | histone deacetylase activity | regulation of transcription | 58% |
| CG33253 | - | - | 55% |
| CG7280 | sulfite oxidase activity | sulfur metabolic process | 53% |
| CG7635 | - | - | 51% |
| Ulp1 | SUMO-specific protease activity | protein processing | 50% |
| PPk17E | protein serine/threonine kinase activity | protein amino acid phosphorrylation | 44% |
| CG4936 | zinc ion binding; nucleic acid binding | - | 40% |
| CG7326 | - | - | 36% |
| Cp110 | - | centriole replication | 35% |
| CG34422 | chromatin binding; DNA binding | phagocytosis, engulfment | 34% |
| CG6361 | serine-type endopeptidase activity. | proteolysis | 33% |
| Sol | calcium-dependent cysteine-type endopeptidase activity | nervous system development | 33% |
| CG17601 | - | - | 32% |
| CG15744 | G-protein coupled receptor activity | G-protein coupled receptor protein signaling pathway | 31% |
| CG1718 | ATPase activity ; transporter activity | - | 31% |
| Lrrk | protein serine/threonine kinase activity | protein amino acid phosphorylation; small GTPase mediated signal transduction | 30% |
| stg1 | - | - | 30% |
| FucTC | fucosyltransferase activity | protein amino acid glycosylation | 28% |
| CG1494 | ATPase activity ; transporter activity | - | 26% |
| CG15745 | - | - | 26% |
| CG33714 | mRNA binding | - | 24% |

**Table S4. Non-neutral mutations predicted by SNAP.**

| Model Category | Gene | Mutation | Expected Accuracy (%) |
|---|---|---|---|
| 1 | CG31220 | F150S | 82 |
| | | V124I | 78 |
| | | T128I | 58 |
| | Rad51D | M184I | 58 |
| 2 | H | A390T | 82 |
| | CG9981 | S809A | 63 |
| | Aats-his | S131L | 63 |
| 3 | CG33714 | D49A | 87 |
| | Dys | L24Q | 70 |
| | | Q1733H | 63 |
| | | P247S | 63 |
| | | A606T | 63 |
| | CG1494 | V154I | 63 |
| | CG15744 | P1707H | 63 |
| | Ulp1 | S1009F | 63 |
| | stg1 | H167L | 58 |
| | CG33253 | P362Q | 58 |
| 4 | Mec2 | L305P | 70 |
| | mei-41 | Q1671H | 70 |
| | Wnt5 | D317E | 58 |
| | CG1640 | T172K | 58 |
| | CG4433 | G91D | 58 |
| | CG11566 | L112V | 58 |

## References


1. Kellenberger C, Leone P, Coquet L, Jouenne T, Reichhart JM, Roussel A: **Structure-function analysis of grass clip serine protease involved in Drosophila Toll pathway activation.** *J Biol Chem* 2011, **286:**12300-12307.
2. Sali A, Blundell TL: **Comparative protein modelling by satisfaction of spatial restraints.** *J Mol Biol* 1993, **234:**779-815.
3. Kubota T, Shiba T, Sugioka S, Furukawa S, Sawaki H, Kato R, Wakatsuki S, Narimatsu H: **Structural basis of carbohydrate transfer activity by human UDP-GalNAc: polypeptide alpha-N-acetylgalactosaminyltransferase (pp-GalNAc-T10).** *J Mol Biol* 2006, **359:**708-727.
4. Huang X, Poy F, Zhang R, Joachimiak A, Sudol M, Eck MJ: **Structure of a WW domain containing fragment of dystrophin in complex with beta-dystroglycan.** *Nat Struct Biol* 2000, **7:**634-638.
